# High-throughput plasma/serum proteomics by coupling nanoparticle protein corona-based sample preparation and capillary zone electrophoresis-tandem mass spectrometry†

**DOI:** 10.1039/d5ay00721f

**Published:** 2025-07-18

**Authors:** Qianyi Wang, Seyed Amirhossein Sadeghi, Fei Fang, Dejin Zheng, Chenxiang Luo, Guangyao Gao, Qianjie Wang, Bin Gu, Liangliang Sun

**Affiliations:** a Department of Chemistry, Michigan State University East Lansing Michigan 48824 USA lsun@chemistry.msu.edu; b Department of Obstetrics Gynecology and Reproductive Biology, Institute for Quantitative Health Science and Engineering, Michigan State University East Lansing Michigan 48824 USA gubin1@msu.edu

## Abstract

A high-throughput workflow for bottom-up proteomics (BUP) of human plasma using capillary zone electrophoresis-tandem mass spectrometry (CZE-MS/MS) and nanoparticle protein corona-assisted sample preparation is presented. The streamlined approach enabled the identification and quantification of hundreds of proteins from plasma/serum samples in 3.5 hours from sample to data. Nanoparticles with varied physiochemical properties studied in this work captured different pools of the plasma/serum proteome in the protein coronas, and the protein corona-based sample preparation approach enabled the measurement of low-abundance proteins compared to the approach without nanoparticles. Applying this high-throughput workflow to serum samples of a mouse NUT carcinoma (NC) cancer model allowed the determination of differentially expressed serum proteins between NC bearing mice and healthy controls. By comparing our quantitative proteomics data with published transcriptomics data, we revealed a handful of potential serum protein biomarkers of NC cancer (*e.g.*, secreted phosphoprotein 1, SPP1). We expect this high-throughput workflow, with additional improvement in the speed of the mass spectrometer, will be useful for advancing the discovery of new protein biomarkers of diseases (*e.g.*, cancer) using plasma/serum samples.

## Abstract 2

A high-throughput workflow for bottom-up proteomics (BUP) of human plasma using capillary zone electrophoresis-tandem mass spectrometry (CZE-MS/MS) and nanoparticle protein corona-assisted sample preparation is presented.

## Graphical abstract

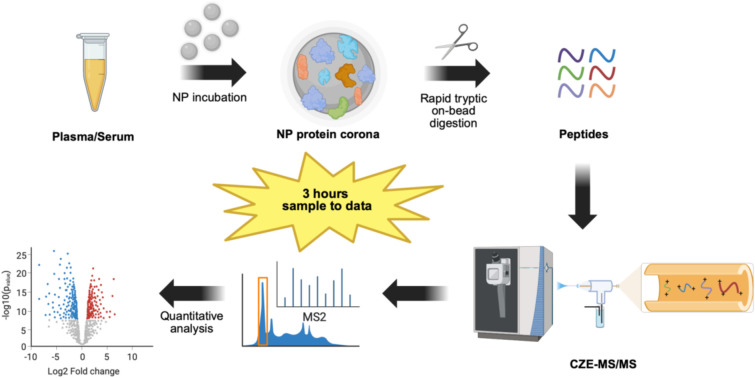

## 1. Introduction

Nanomedicine has gained significant interest in pharmaceutical research due to the promising capability of drug targeting and drug delivery.^1–6^ The therapeutic efficacy, targeting ability, toxicity, cellular interactions, and biodistribution of nanoparticle-bound medicine are heavily influenced by the formation of the biomolecule corona, *i.e.*, protein corona.^7–10^ Nanoparticle protein corona refers to a layer of proteins that are naturally attached to the surface of nanoparticles (NPs) when they enter a biological environment, such as blood or other body fluids.^11–13^ It has been well established that the distinct profiles of nanoparticle protein corona layers can not only reflect the complex thermodynamics, kinetics, and biological interactions of NPs but also provide a snapshot of the proteome information.^13–18^ Therefore, studying the composition of the protein corona has the potential to reveal the biological identity of NPs and provide insights into the proteome of the surrounding biological environment.

Blood plasma plays a central and integrative role in human physiology, acting as a universal reflection of an individual's state or phenotype for disease diagnosis and therapeutic monitoring.^19^ However, plasma proteomics is challenging as the broad dynamic range of protein abundance in plasma remains the major difficulty.^20^ To overcome this issue, one of the emerging applications is using nanoparticle protein corona to reduce blood plasma proteome complexity and protein concentration dynamic range, facilitating the detection and identification of low-abundance disease-associated biomarkers.^21–28^ The antibody-based approach has been utilized in human plasma/serum proteomics for years to deplete high-abundance proteins.^29^ One advantage of the protein corona approach compared to the antibody method is that protein corona depletes high-abundance proteins and enriches low-abundance proteins at the same time. Thousands of human plasma proteins can be identified using the protein corona approach in a single experiment,^21,25,30^ which is drastically higher than the best dataset from the antibody approach. This is the main reason for focusing on the protein corona approach in this work.

Mass spectrometry (MS)-based proteomics is broadly recognized as an effective approach for characterizing the protein corona, allowing for the precise identification and quantification of proteins/proteoforms adsorbed on nanoparticle surfaces.^21–23,31^ MS-based bottom-up proteomics (BUP) is more broadly used for protein corona study and plasma/serum proteomics because of its much better sensitivity.^26^ However, the throughput remains a significant challenge in BUP-based plasma studies because of the time-consuming steps in the typical BUP workflow from sample preparation, protein digestion, to LC-MS. Thus, the development of a high-throughput BUP workflow for the biomarker discovery in plasma proteomics is essential. Shen *et al.* demonstrated comparable numbers of protein identifications (IDs) from 15 min of immobilized trypsin digestion and 12 h of free trypsin digestion of mouse brain tissue sample, proving the application of rapid tryptic digestion for high-throughput BUP by NPs.^32^ Capillary zone electrophoresis (CZE)-tandem MS (MS/MS) has been widely acknowledged as a valuable technique for BUP, such as highly sensitive analysis of mass-limited biological samples, analysis of disease-related biomarkers, large-scale quantitative analysis, and many others.^33–38^ CZE is a high-efficiency separation method based on an analyte's electrophoretic mobility. The use of shorter capillaries can accelerate CZE separations to only several minutes or even less than a minute,^39–41^ making it a promising approach for high-throughput proteomics.

In this work, we developed a high-throughput BUP workflow for plasma/serum analysis by coupling NP protein corona, rapid on-bead tryptic digestion, and CZE-MS/MS, identifying hundreds of proteins from plasma/serum samples in 3.5 hours of total analysis time from sample to data. For the first time, we performed quantitative BUP analysis of mouse serum samples of NUT carcinoma using one of the first genetically engineered mouse models of NUT carcinoma,^42^ discovering novel potential cancer biomarkers.

## 2. Experimental section

### 2.1. Ethical statement

All mouse studies here were performed at Michigan State University (MSU) following local laws and guidelines of the MSU Campus Animal Resources (CAR) and Institutional Animal Care and Use Committee (IACUC). CAR and IACUC oversee and evaluate animal studies and set guidelines for housing and breeding, sacrifice, and other manipulations. CAR animal facilities are AAALAC accredited. All animal work in this study has been approved by the MSU CAR and IACUC in AAALAC credited facilities under two procedures, PROTO202000143 and PROTO 202300127.

### 2.2. Mouse line and breeding

Mice from the NUT Carcinoma Translocator (NCT) strain CD1-Brd4^em1Gumsu^Nutm^1em1Gumsu^/Mmmh were obtained from an in-house breeding colony. These mice were crossed with the *Krt14Cre* strain to generate oral NC bearing mice.^43^ Tumor growth were monitored by bioluminescent imaging (BLI).

### 2.3. Materials and reagents

Amine-terminated NPs (Catalog #BP617) and carboxylate-terminated NPs (Catalog #BP618) were purchased from Bangs Laboratories, Inc. (Fishers, IN). Single-pot solid-phase-enhanced sample preparations (SP3) hydrophilic NPs (Catalog #45152105050250) and SP3 hydrophobic NPs (Catalog #65152105050250) were obtained from Cytiva (Marlborough, MA). Dulbecco's Phosphate-Buffered Saline (DPBS, 1×), sodium dodecyl sulfate (SDS), ammonium bicarbonate (ABC), and dithiothreitol (DTT) were from Sigma-Aldrich (St. Louis, MO). Plain polystyrene NPs were obtained from Polysciences (http://www.polysciences.com). Trypsin (bovine pancreas TPCK-treated), formic acid (FA), acetonitrile (ACN), methanol, LC/MS grade water, and bicinchoninic acid (BCA) assay kit were purchased from Fisher Scientific (Pittsburgh, PA). The protein LoBind tube was from Eppendorf (Enfield, CT). Healthy human plasma protein was purchased from Innovative Research (http://www.innov-research.com) and diluted to 55% using 1× DPBS. Biological triplicates of healthy and NUT cancer mouse serum (healthy: BN010, BN012, and BN110; NUT cancer: KBN002, KBN010 and KBN111).^42^*Krt14Cre*-driven NUT carcinoma mouse models were generated as described by crossing the *Krt14Cre* mouse line with the NUT Carcinoma Translocator mouse line (MMRRC 071753-MU).^42^ Serum was sampled from end-stage tumor-bearing mice and healthy controls following the retro-orbital blood collection procedure as previously described.^44^

### 2.4. Formation of nanoparticle protein corona

For human plasma nanoparticle protein corona, 1.25 mg amine-terminated magnetic NPs, carboxylate-terminated magnetic NPs, SP3 hydrophilic magnetic NPs, SP3 hydrophobic magnetic NPs or polystyrene NPs were individually washed with 200 μL water twice, and then incubated with 1 mL 55% human plasma at 37 °C for 1 h with constant stirring at 350 rpm to form nanoparticle protein corona. A magnet rack was used to separate the solution for four magnetic NPs, while centrifugation at 14 000*g* for 20 min was applied to remove the solution for polystyrene NPs. Next, the nanoparticle protein coronas were washed with 500 μL ice-cold DPBS twice, followed by 500 μL ice-cold water twice. The resulting nanoparticle protein coronas in the solid phase were collected for further BUP sample preparation. For SDS-PAGE evaluation of the performances by different nanoparticle protein coronas, the proteins from nanoparticle protein coronas were eluted with 100 μL 0.2% SDS in water. The protein concentrations in the eluates were measured by BCA assay. Fifteen μg eluted protein was loaded into each lane of SDS-PAGE gel for analysis.

For mouse serum nanoparticle protein corona, 200 μg amine-terminated magnetic NPs, and carboxylate-terminated magnetic NPs were individually washed with 200 μL water twice and then incubated with 40 μL 55% mouse serum at 37 °C for 1 h with constant stirring at 350 rpm to form nanoparticle protein corona. A magnet rack was used to separate the solution for four magnetic NPs, while centrifugation at 14 000*g* for 20 min was applied to remove the solution for polystyrene NPs. Next, the nanoparticle protein coronas were washed with 20 μL ice-cold DPBS twice, followed by 20 μL ice-cold water twice. The resulting nanoparticle protein coronas in the solid phase were collected for further BUP sample preparation.

### 2.5. Sample preparation for BUP

The resulting nanoparticle protein coronas in the solid phase from the “Formation of nanoparticle protein corona” section (∼15 μg of total proteins) was dispersed in 15 μL of 100 mM ABC buffer (pH 8.0) containing 5 mM DTT and led the protein corona to be fully denatured and reduced at 90 °C for 15 min. Then, the protein corona was cooled down to room temperature, followed by trypsin (3 μg) digestion at 37 °C for 1 h. The digestion was finally terminated by adding formic acid (0.6% (v/v) final concentration), and the supernatants were collected in the LoBind tubes. To fully elute the peptides, 10 μL 20% ACN in 100 mM ABC was incubated with the nanoparticle at 37 °C for 10 min and combined with the supernatant from the previous portion for downstream CZE-MS/MS analysis.

### 2.6. CZE-MS/MS analysis

Linear polyacrylamide (LPA)-coated fused silica capillaries (50 μm i.d., 360 μm o.d.) were prepared according to our previous studies.^45,46^ The CZE-MS/MS system configuration involved the integration of a CESI 8000 Plus CE system (Beckman Coulter) with an Orbitrap Exploris 480 mass spectrometer (Thermo Fisher Scientific), employing an in-house-built electrokinetically pumped sheath-flow CE-MS nanospray interface.^47,48^ The interface featured a glass spray emitter pulled using a Sutter P-1000 flaming/brown micropipette puller to achieve an orifice size of 30–35 μm, filled with sheath buffer composed of 0.2% (v/v) formic acid and 10% (v/v) methanol. The spray voltage was about 2 kV. The length of the LPA-coated CZE capillary was 80 cm. The capillary inlet was securely affixed within the cartridge of the CE system, while its outlet was inserted into the emitter of the interface. The capillary outlet to emitter orifice distance was maintained at approximately 0.5 mm. Fifty nL (∼30 ng) of each corona peptide sample was loaded for CZE-MS/MS. Following this, the capillary inlet was filled with the background electrolyte (BGE, 5% (v/v) acetic acid), initiating the CZE separation process under a separation voltage of 30 kV, and the separation time was 50 min. After the separation, 30 kV voltage and 15 psi pressure were applied for 10 min to clean up the capillary.

For the mass spectrometer, all experiments were conducted using an Orbitrap Exploris 480 mass spectrometer (Thermo Fisher Scientific) in data-dependent acquisition (DDA) mode. Ion transfer tube temperature was set at 320 °C and peptide mode was enabled. Pressure mode was set as standard. Full MS scans were acquired in the Orbitrap mass analyzer over the *m*/*z* 300–1500 range with a resolution of 60 000 (at 200 *m*/*z*). Normalized AGC targets for MS and MS/MS were set at 300% and 150%, respectively. Only precursor ions with an intensity exceeding 1 × 10^5^ and a charge state between 2 and 7 were fragmented in the higher-energy collisional dissociation (HCD) cell and analyzed by the Orbitrap mass analyzer with a resolution of 7500 (at 200 *m*/*z*). The numbers of dependent scans for human plasma and mouse serum were at 15 and 25, respectively. Monoisotopic peak determination was set to peptide and the option “Relax restrictions when too few precursors are found” was checked. One microscan was used for both MS and MS/MS. The normalized collision energy was set at 30%. The maximum ion injection times for MS and MS/MS spectra acquisition were both set as auto. The precursor isolation width was 1.4 *m*/*z*. The first mass for MS/MS was set at *m*/*z* 100. The dynamic exclusion was applied with a duration of 15 s, and the exclusion of isotopes was enabled. The mass spectrometry proteomics data have been deposited to the ProteomeXchange Consortium *via* the PRIDE^49^ partner repository with the dataset identifier PXD063043.

### 2.7. Database search

For human plasma nanoparticle protein corona, database searching of the raw files was performed in Proteome Discoverer 2.2 with SEQUEST HT search engine against the UniProt proteome database of human (UP000005640, 82 697 entries, version 12/2023). Database searching of the reversed database was also performed to evaluate the false discovery rate (FDR). Search parameters included full tryptic digestion, allowing up to 2 missed cleavages, with precursor mass tolerance set to 20 ppm and fragment mass tolerance to 0.05 Da. Acetyl (protein N-term) and phospho (S, T, Y) were set as variable modifications. Data was filtered with a peptide-level FDR of 1%, and protein grouping was applied. The human plasma protein identification lists by different nanoparticle protein coronas are shown in the ESI I.†

For mouse serum nanoparticle protein corona, raw data files were analyzed using MaxQuant software (Version 2.1.2.0) with the Andromeda search engine against the UniProt proteome database of mouse (UP000000589, 54 707 entries, version 02/2024).^50^ The peptide mass tolerances for the initial and main searches were set to 20 ppm and 4.5 ppm, respectively, with a fragment ion mass tolerance of 20 ppm. Trypsin was specified as the protease, and dynamic modifications included oxidation on methionine and acetylation at the protein N-terminus. Label-free quantification (LFQ) option was checked and the LFQ minimum ratio count was at 2. Intensity-based absolute quantification (iBAQ) was checked to report the measure of protein abundance. The minimum peptide length was set to 7 amino acids, and FDRs were controlled at 1% for both peptides and proteins. The mouse serum protein identification lists and the LFQ information by different nanoparticle protein coronas are shown in the ESI II.†

### 2.8. Statistical analysis

For LFQ of amine-terminated nanoparticle and carboxylate-terminated nanoparticle treated mouse serum (healthy and nut cancer), each sample contains technical duplicate CZE-MS/MS runs. Twelve MS raw files for each nanoparticle were applied for statistical analysis, and iBAQ values were used as the absolute abundance. The quantitative results were further analyzed using Perseus software.^51^ The intensities of each protein were log2 transformed, and the significantly differentially expressed proteins were determined by performing *t*-test analysis using the Perseus software to generate ‘−log(*P*-value)’ and ‘log2(fold change of KBN to BN)’. *P*-Value at 0.05 and fold change at 2 (log_2_(KBN/BN) = 1) were used for making volcano plots by DataGraph software (Version 5.3).

## 3. Results and discussion

### 3.1. A high-throughput BUP workflow of CZE-MS/MS using human plasma

**Fig. 1:**
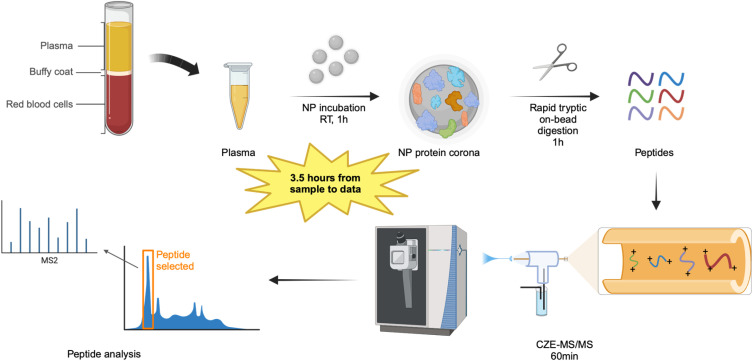
Schematic of the MS-based BUP workflow for magnetic nanoparticle protein corona using amine-terminated NPs, carboxylate-terminated NPs, SP3 hydrophilic NPs, SP3 hydrophobic NPs, a human plasma sample, and CZE-MS/MS.

To develop an effective BUP workflow for high-throughput analysis of human plasma, we tested four types of magnetic NPs including amine-terminated NPs, carboxylate-terminated NPs, SP3 hydrophilic NPs, and SP3 hydrophobic NPs. We used a commercialized healthy human plasma and CZE-MS/MS for peptide separation, detection, and identification. Fig. 1 describes the detailed BUP workflow, which takes approximately 3.5 hours from sample preparation to generate MS raw files. The full procedures are outlined in the experimental section. Briefly, 55% human plasma was first incubated with magnetic NPs for 1 hour to form a nanoparticle protein corona, followed by washing steps to remove unbound proteins. The nanoparticle protein corona underwent rapid on-bead tryptic digestion for 1 hour, with hydrophilic peptides remaining in the aqueous phase. To ensure complete peptide elution, the NPs were incubated with an elution buffer containing 20% ACN. Finally, the second eluted peptides were combined with the initial aqueous phase and directly subjected to a 1 hour CZE-MS/MS analysis without any additional processing.

### 3.2. Validation of the high-throughput BUP workflow using human plasma

**Fig. 2:**
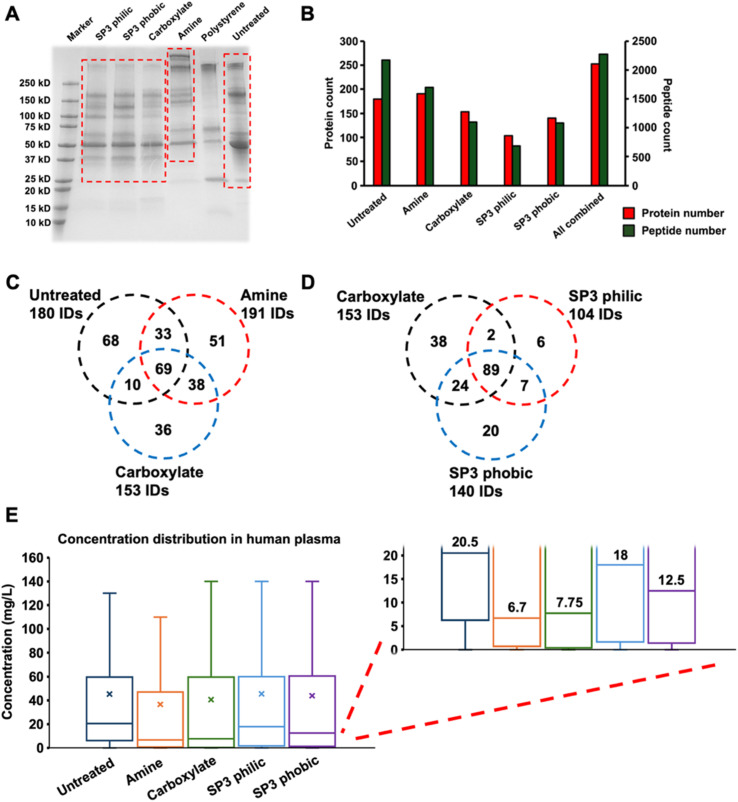
Different protein profiles revealed by amine-terminated NPs (Amine), carboxylate-terminated NPs (Carboxylate), SP3 hydrophilic NPs (SP3 philic), SP3 hydrophobic NPs (SP3 phobic), polystyrene NPs (Polystyrene) and untreated human plasma. (A) SDS-PAGE data of nanoparticle protein coronas and untreated human plasma. (B) The protein and peptide identification count by different nanoparticle protein coronas and untreated human plasma. (C) Protein overlaps among amine-terminated NPs (191 protein IDs), carboxylate-terminated NPs (153 protein IDs), and untreated human plasma (180 protein IDs). (D) Protein overlaps among carboxylate-terminated NPs (153 protein IDs), SP3 hydrophilic NPs (104 protein IDs) and SP3 hydrophobic NPs (140 protein IDs). (E) Protein concentration distribution by different nanoparticle protein coronas and untreated human plasma, and all the numbers are at the unit of mg L^−1^. The protein abundance information was obtained from the Human Protein Atlas database.

Prior to CZE-MS/MS, we employed a preliminary SDS-PAGE analysis (Fig. 2A) to compare the profiles of the untreated human plasma, four different magnetic nanoparticle protein coronas, and a non-magnetic polystyrene NPs, which has demonstrated the robustness for forming protein corona of human plasma.^31^ Among four magnetic NPs, SP3 hydrophilic NPs and SP3 hydrophobic NPs are well developed for rapid, robust, and efficient protein sample processing for BUP.^52^ Instead of the tryptic cleavage, the intact proteins were eluted from the nanoparticle surface by an elution buffer containing SDS. The SDS-PAGE result showed that the major protein bands (37–150 kDa) from carboxylate-terminated NPs are relatively consistent with those from SP3 hydrophilic NPs and SP3 hydrophobic NPs. It must be noted that both SP3 NPs contain a carboxyl group covalently bound on the nanoparticle surface, leading to a similar surface chemistry to carboxylate-terminated NPs of forming protein corona. However, the protein profiles from carboxyl group-based NPs, amine-terminated NPs, and polystyrene NPs are all significantly different from each other, representing the distinct pools of protein coronas by different NPs. Furthermore, all NPs performed obvious drops at the intense bands compared to the untreated human plasma, such as near-150 kDa (possibly IgGs) and near-50 kDa (possibly α-1-antitrypsin, haptoglobin or plasminogen) bands, showing the effective depletion of high-abundance proteins in human plasma. Next, CZE-MS/MS was applied to deeply explore the protein corona profiles by different NPs. Fig. 2B depicts the protein and peptide identification numbers (IDs) from untreated human plasma and four magnetic nanoparticle protein coronas. Amine-terminated NPs achieved slightly higher protein IDs (191 IDs) than untreated human plasma (180 IDs) with fewer peptide IDs (2000 IDs *vs.* 2172 IDs), while all carboxyl group-based NPs showed lower protein IDs (153, 104, and 140 IDs for carboxylate-terminated NPs, SP3 hydrophilic NPs and SP3 hydrophobic NPs, respectively) and peptide IDs (1134, 703 and 1117 IDs for carboxylate-terminated NPs, SP3 hydrophilic NPs and SP3 hydrophobic NPs, respectively). This revealed individual NPs didn't perform a significant increase in the protein or peptide IDs compared to untreated human plasma. However, by combining four different NPs, we could attain much higher protein IDs (253 IDs) with comparable peptide IDs (2272 IDs) to untreated human plasma, showing the potential of coupling distinct NPs for protein corona to reach higher protein IDs.

Interestingly, the untreated human plasma, amine-terminated NPs, and carboxylate-terminated NPs produced different protein profiles, evidenced by the low protein identification overlap among them (Fig. 2C). This can be explained by the distinct functional groups on the nanoparticle surface to selectively capture different proteins. On the contrary, a high protein identification overlap was found within three carboxyl group-based NPs (Fig. 2D), which is consistent with the similar protein band distributions in the previous SDS-PAGE result. We then speculated that the proteins identified in the untreated human plasma contained many high-abundance proteins such as albumin and immunoglobulin, which are also identified from each magnetic NPs as the overlap. However, for the proteins identified from each nanoparticle protein corona, there are still a good amount of non-overlapped proteins identified and we assumed the nanoparticle protein corona could significantly decrease the concentration dynamic range of human plasma. To prove this hypothesis, we checked the identified proteins' concentrations by untreated human plasma and four nanoparticle protein coronas from the blood protein section in the Human Protein Atlas database.^53^ As shown in Fig. 2E, the protein concentration distribution data demonstrated the benefits of NP protein corona for reducing the concentration dynamic range to detect more low-abundance proteins in human plasma, verified by the lower median value from different NPs (6.7, 7.75, 18, and 12.5 mg L^−1^) compared to untreated human plasma (20.5 mg L^−1^). In addition, we revealed that 9.2%, 38.3%, 46.6%, 29.7% and 35.4% of the identified proteins from the untreated human plasma and treated samples by amine-terminated NPs, carboxylate-terminated NPs, SP3 hydrophilic NPs, and SP3 hydrophobic NPs, have concentrations lower than 1 mg L^−1^. The results suggest that NPs enrich low-abundance proteins, thereby facilitating their identification within the complex blood proteome. Considering the low protein overlaps from NPs with different functional groups and the capability of detecting more low-abundance proteins, we decided to apply only amine-terminated NPs and carboxylate-terminated NPs as the representative NPs for further experiments.

### 3.3. Application of the high-throughput BUP workflow to healthy and NUT cancer mouse serum samples for potential protein biomarker discovery

**Fig. 3:**
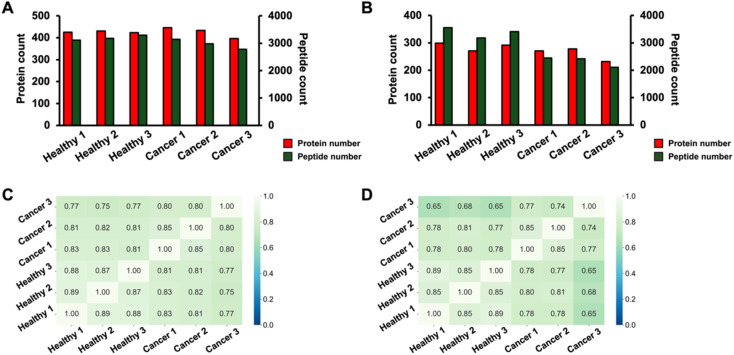
Protein and peptide identification counts and protein overlaps by amine-terminated NPs (A and C), carboxylate-terminated NPs (B and D) using serum from healthy mouse model (Healthy 1, 2 and 3 represent for BN010, BN012 and BN110, respectively) and NC mouse model (Cancer 1, 2 and 3 represent for KBN002, KBN010 and KBN111, respectively). (A) The protein and peptide identification counts by amine-terminated NPs. (B) The protein and peptide identification counts by carboxylate-terminated NPs. (C) Protein overlaps by amine-terminated NPs. (D) Protein overlaps by carboxylate-terminated NPs.

NUT carcinoma (NC) is an aggressive cancer characterized by chromosomal rearrangements, typically involving the fusion of the NUTM1 gene with genes like BRD4, leading to uncontrolled cellular growth and blocked differentiation.^54^ This rare carcinoma occurs primarily in midline structures such as the head, neck, and mediastinum, affecting both children and adults, with a poor prognosis despite intense treatment.^55^ Research is actively exploring the origins and epigenetic mechanisms of NC, aiming to develop more effective therapeutic strategies and early-stage detection. Mouse serum proteomics can be used to study disease models to identify potential protein biomarkers of NC. We recently developed a genetically engineered NC mouse model that faithfully replicates NC oncogenesis in a controlled experimental setting with the precise regulation of key parameters.^42^ Here, we applied the proteomics workflow to serum samples from this NC mouse model for potential protein biomarker discovery. Three biological replicates of mouse serum from the healthy and oral NC mice were used in the study. Amine-terminated and carboxylate-terminated nanoparticle protein coronas were employed for the sample preparation. Fig. 3A and B display that amine-terminated nanoparticle protein corona outperforms carboxylate-terminated nanoparticles in terms of the average number of protein IDs (426 *vs.* 274) and peptide IDs (3081 *vs.* 2853) from biological triplicates, agreeing well with our previous human plasma data mentioned above. Amine-terminated nanoparticle protein corona approach also showed a consistent number of protein IDs across the biological triplicate with a 4% relative standard deviation (RSD). Carboxylate-terminated nanoparticle protein corona produced relatively consistent identification of proteins with an RSD of 9%. We further studied the protein overlaps between the biological replicates of healthy and cancer samples for amine and carboxylate nanoparticles, Fig. 3C and D. The overlap within the same sample types (healthy against healthy, and cancer against cancer) averages at 0.85 ± 0.04 for amine-terminated NPs and at 0.83 ± 0.04 for carboxylate-terminated NPs, while the overlap within different sample types (healthy against cancer) shows an average at 0.80 ± 0.03 for amine-terminated NPs and at 0.74 ± 0.06 for carboxylate-terminated NPs. The data suggest that two NPs can both perform nanoparticle protein corona efficiently with good reproducibility, allowing for the confident quantitative analysis of proteins.

**Fig. 4:**
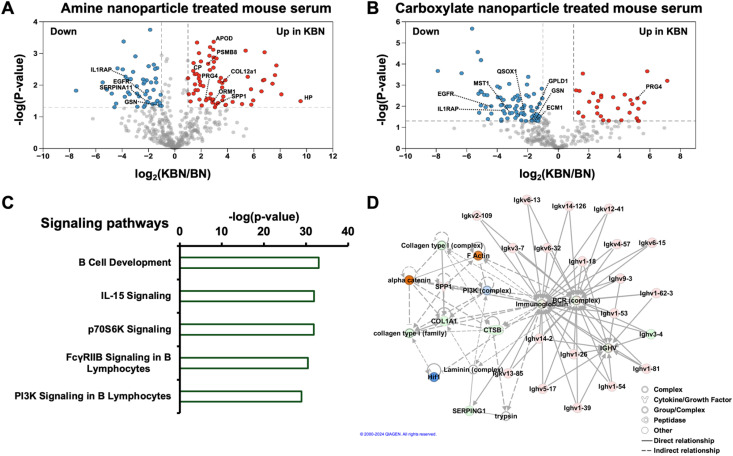
LFQ of healthy mouse serum model (BN) and NC mouse serum model (KBN). Volcano plot of LFQ of amine-terminated nanoparticle-treated mouse serum (A) and carboxylate-terminated nanoparticle-treated mouse serum (B). Up-regulated biomarkers in the NC mouse serum model are labeled in red, while down-regulated ones are marked in blue. Examples of some genes related to candidate biomarkers of negative survival in the tumors are marked. (C) An ingenuity pathway analysis reported some cancer, organismal injury, and abnormalities diseases that are related to the differentially expressed genes in the two mouse serum models by amine-terminated NPs. (D) Proteins with significant abundance differences within two mouse serum models correspond to genes that are involved in cancer-related networks with high scores. Those genes are highlighted in pink (increased), green (decreased), orange (predicted activation), and blue (predicted inhibition). Copyright permission has been granted by QIAGEN for using the network data.

Next, LFQ was applied for both amine-terminated nanoparticle-treated and carboxylate-terminated nanoparticle-treated mouse serum data. The volcano plots in Fig. 4A and B show the up-regulated (red) and down-regulated (blue) proteins by a protein LFQ intensity ratio cutoff of 2 (cancer/control) and *p*-value at 0.05 in the amine-terminated nanoparticle-treated and carboxylate-terminated nanoparticle-treated NC mouse serum model, respectively. Overall, 116 of 489 proteins and 111 from 332 proteins had statistical differences in abundance between the healthy and NC mouse serum for amine-terminated NPs and carboxylate-terminated NPs, respectively. Sixty-seven proteins were up-regulated and 49 proteins were down-regulated in NC mouse serum by amine-terminated NPs, while 31 proteins were up-regulated and 80 proteins were down-regulated in NC mouse serum by carboxylate-terminated NPs. We further evaluated the quantitative consistency of the 44 overlapping proteins identified in both NP conditions, observing a strong linear correlation of LFQ intensity between the two NPs (Pearson's *r* of 0.9). In total, combining the data from two different nanoparticles, 183 differentially expressed proteins were determined. This further showed the advantage of coupling different types of nanoparticle protein corona to boost the number of differentially expressed proteins. Furthermore, according to the published transcriptomic data (GSE263558 (https://www.life-science-alliance.org/lookup/external-ref?link_type=NCBIGEO&access_num=GSE263558&atom=%2Flsa%2F7%2F7%2Fe202402602.atom)),^42^ we found 15 genes (up-regulated : down-regulated = 11 : 4 in NC mouse serum) from amine-terminated NPs and 8 genes (up-regulated : down-regulated = 1 : 7 in NC mouse serum) from carboxylate-terminated NPs to be candidate biomarkers of negative survival in the tumors. In total, 19 genes (Table 1) were revealed to be potential biomarkers by combining two NPs' data, suggesting that different NPs could be used as complementary tools to identify more biomarkers from serum samples. Among the 19 genes, Spp1 is a confident gene associated with aggressive cancers, produced in various organs and found in body fluids such as serum and urine. Spp1 is expressed in specific cell types, including osteoblasts, macrophages, and immune cells, and is also present in cancer cells, with elevated levels of SPP1 correlating with poor prognosis in several cancers.^56–58^ SPP1 promotes cancer cell growth and resistance to chemoradiotherapy through the induction of epithelial–mesenchymal transition, autophagy, and metabolic alterations, primarily *via* activation of the PI3K/Akt and MAPK pathways.^59^

**Table 1:** Summary of cancer-related protein biomarkers identified by nanoparticle protein corona

Gene	Protein name	Amine-terminated nanoparticle protein corona	Carboxylate-terminated nanoparticle protein corona	Up or down regulated in NC mouse model serum
Spp1	Secreted phosphoprotein 1	×		Up
Col12a1	Collagen, type XII, alpha 1	×		Up
Hp	Haptoglobin	×		Up
Apod	Apolipoprotein D	×		Up
Orm1	Alpha-1-acid glycoprotein 1	×		Up
Psmb8	Proteasome subunit beta type-8	×		Up
Prg4	Proteoglycan 4	×	×	Up
Lrg1	Leucine-rich HEV glycoprotein	×		Up
Cp	Ferroxidase	×		Up
Ctla2a	Protein CTLA-2-alpha	×		Up
Lbp	Lipopolysaccharide-binding protein	×		Up
Gsn	Gelsolin	×	×	Down
Serpina11	Serpin A11	×		Down
Egfr	Epidermal growth factor receptor	×	×	Down
Il1rap	Interleukin-1 receptor accessory protein	×	×	Down
Ecm1	Extracellular matrix protein 1		×	Down
Gpld1	Phosphatidylinositol-glycan-specific phospholipase D		×	Down
Qsox1	Sulfhydryl oxidase 1		×	Down
Mst1	Macrophage stimulating 1 (hepatocyte growth factor-like)		×	Down

We then performed an ingenuity pathway analysis (IPA) of the genes with differential abundance between healthy and NC mouse serum models using amine-terminated NPs as an example. The genes are involved in cancer-related pathways such as immune-related pathways (B cell development, IL-15 signaling, and FcγRIIB signaling), cell growth pathways (p70S6K signaling), and proliferation and migration pathways (PI3K signaling), Fig. 4C. IPA network analysis revealed that 20 proteins (highlighted in red) showed higher abundance and 4 proteins (highlighted in green) showed lower abundance in the NC mouse serum model compared to the healthy mouse serum model involving cancer, organismal injury, and abnormality-related network (score, 57), Fig. 4D. Those proteins belong to several groups such as growth factor (*e.g.*, SPP1), peptidase (*e.g.*, CTSB), complex (*e.g.*, Collagen type i or I) and others, and the proteins all have direct (solid line) and indirect (dotted line) interactions with one another. The abundance changes of proteins in the serum could potentially reflect the activities of proteins in the cell, *e.g.*, PI3K complex. PI3K (phosphatidylinositol 3-kinase, located in the cytoplasm) intracellular pathway is critical in regulating cell growth, survival, and metabolism, and the PI3K mutations activate the downstream AKT/mTOR signaling cascade, which promotes tumorigenesis by enabling cells to evade apoptosis, enhance proliferation, and develop resistance to conventional therapies.^60^ In the IPA network, we noticed that up-regulated proteins like SPP1 and down-regulated proteins like collagen type I, collagen type i, and COL1A1 in the NC mouse cancer model have indirect interaction (dotted line) with PI3K, which is predicted to indirectly activate proteins located in cytoplasm like alpha-catenin and F Actin. We also noted that PI3K has indirect interaction (dotted line) with immunoglobulin BCR complex which directly interact with many up-regulated immunoglobulin variables found from our experimental results. All the differentially expressed proteins associated with PI3K and PI3K-affected proteins could be used as potential biomarkers for cancer, organismal injury, and abnormality.

## 4. Conclusions

In this study, we have developed a high-throughput nanoparticle protein corona-based plasma/serum proteomics workflow, completing the whole workflow from sample to data within 3.5 hours. Our results demonstrated the effectiveness of this approach in reducing sample complexity and increasing the identification of low-abundance proteins, addressing the inherent challenges in plasma proteomics. For the first time, we performed quantitative BUP analysis of mouse serum samples of NUT carcinoma using one of the first genetically engineered mouse models of NUT carcinoma, discovering novel potential cancer biomarkers. The results demonstrate the high potential of our high-throughput workflow for large-scale plasma proteomics for discovering novel biomarkers of diseases.

Some limitations need to be addressed in our future work. Firstly, the current capillary length employed for CZE-MS/MS is 80 cm, resulting in an analysis time of approximately 1 hour. The separation could be much faster with shorter capillaries (*e.g.*, 30 cm) to improve throughput, which requires a minimized commercial CE system. Secondly, the scanning rate of Orbitrap-based mass spectrometer is limited, causing a relatively lower number of protein IDs compared to other fast mass spectrometers like trapped ion mobility spectrometry-time-of-flight (TIMS-TOF) or Orbitrap Astral mass spectrometers.^61–63^ Thirdly, the robustness of CZE-MS/MS for large-scale plasma/serum proteomics applications still needs to be evaluated and compared with the commonly used LC-MS/MS approach. Overall, we expect that the combination of nanoparticle protein corona-based sample preparation, fast and robust CZE separation with a short separation capillary, and an ultra-fast mass spectrometer will advance the field of plasma proteomics for biomarker discovery and disease diagnosis substantially. We also expect that employing data-independent acquisition (DIA) in our workflow will further enhance the proteome coverage of plasma and serum samples.

## Conflicts of interest

The authors declare no competing financial interest.

## Supplementary Material

AY-017-D5AY00721F-s001

AY-017-D5AY00721F-s002

## Data Availability

The data supporting this article have been included as part of the ESI.† The MS raw data and processed data have also been deposited to the ProteomeXchange Consortium *via* the PRIDE^64^ partner repository with the dataset identifier PXD063043.
